# Intraoperative Conversion to ALPPS in a Case of Intrahepatic Cholangiocarcinoma

**DOI:** 10.1155/2015/273641

**Published:** 2015-11-16

**Authors:** F. Oldhafer, K. I. Ringe, K. Timrott, M. Kleine, W. Ramackers, S. Cammann, M. D. Jäger, J. Klempnauer, H. Bektas, F. W. R. Vondran

**Affiliations:** ^1^Regenerative Medicine & Experimental Surgery (ReMediES), Department of General, Visceral and Transplant Surgery, Hannover Medical School, 30625 Hannover, Germany; ^2^Department of Diagnostic and Interventional Radiology, Hannover Medical School, 30625 Hannover, Germany

## Abstract

*Background*. Surgical resection remains the best treatment option for intrahepatic cholangiocarcinoma (ICC). Two-stage liver resection combining* in situ* liver transection with portal vein ligation (ALPPS) has been described as a promising method to increase the resectability of liver tumors also in the case of ICC.* Presentation of Case*. A 46-year-old male patient presented with an ICC-typical lesion in the right liver. The indication for primary liver resection was set and planed as a right hepatectomy. In contrast to the preoperative CT-scan, the known lesion showed further progression in a macroscopically steatotic liver. Therefore, the decision was made to perform an ALPPS-procedure to avoid an insufficient future liver remnant (FLR). The patient showed an uneventful postoperative course after the first and second step of the ALPPS-procedure, with sufficient increase of the FLR. Unfortunately, already 2.5 months after resection the patient had developed new tumor lesions found by the follow-up CT-scan.* Discussion*. The presented case demonstrates that an intraoperative conversion to an ALPPS-procedure is safely applicable when the FLR surprisingly seems to be insufficient.* Conclusion*. ALPPS should also be considered a treatment option in well-selected patients with ICC. However, the experience concerning the outcome of ALPPS in case of ICC remains fairly small.

## 1. Introduction

Intrahepatic cholangiocarcinoma (ICC) is the second most common primary liver tumor representing about 10% to 20% of all primary malignant liver tumors [1, 2]. However, recent reports have documented an increase in the incidence of ICC, in particular in the well developed countries of the western hemisphere. Particularly primary sclerosing cholangitis has been identified as a major risk factor for development of ICC [3, 4]. Further known risk factors are chronic hepatitis C or HIV infection, liver cirrhosis, diabetes mellitus, and chronic inflammatory bowel disease [5]. Patients typically remain asymptomatic for a long period and ICC mostly occurs in the fifth or sixth decade of life. Surgical resection remains the best treatment option for ICC concerning patient's long-term survival [6]. Our group showed that patients after R0-resection show an overall survival of 67% after 1 and 40% after 3 years, respectively. In comparison, patient's survival without resection is only 26% and 3.4% after 1 and 3 years, respectively [6]. Therefore, patients with ICC may benefit even from extended liver surgery. Unfortunately, resectability rates for ICC only range between 46% and 75% [7]. The recently developed strategy to perform a two-stage liver resection combining* in situ* liver transection with portal vein transection, also known as the ALPPS-procedure (Associating Liver Partition and Portal vein ligation for Staged hepatectomy), has been described as a promising method to increase the resectability of marginally resectable or locally unresectable liver tumors [8]. Since the introduction of ALPPS, several groups worldwide have adopted this new technique mainly to enlarge the pool of patients with resectable colorectal liver metastasis [9]. However, the ALPPS-procedure is also of particular interest for ICC since surgical therapy offers the only chance of cure in these patients.

## 2. Presentation of Case

A 46-year-old man presented in our outpatient clinic with a suspicious lesion in the right liver. The patient had no symptoms or discomfort before admission. He denied any weight loss, fever, or pain. The patient had a known ulcerative colitis (UC) which was under medication with Azathioprine and Budesonide. One and a half years in advance he was already admitted to the hospital with elevated liver enzymes and the strong suspicion of a primary sclerosing cholangitis. However, this diagnosis could not be confirmed in a liver biopsy and liver enzymes normalized after a few weeks. Apart from the known UC he had no past medical history or took any medication. There was no family history of liver conditions or gastrointestinal carcinomas. On physical examination there were no abnormalities. The CT-scan showed an ICC-typical lesion in segments V and VIII of the liver. Liver biopsy confirmed the diagnosis of ICC. Preoperative staging CT-scan (incl. chest CT-scan; performed 24 days prior to surgery) showed no extrahepatic tumor manifestations. Further, serum tumor markers CEA and CA 19-9 were significantly elevated with 40 *μ*g/L and 2569 kU/L, respectively. Consequently, indication for liver resection was set and planed as right hepatectomy. Informed consent obtained from the patient nevertheless also included performance of an extended right hepatectomy and ALPPS.

**Figure 1:**
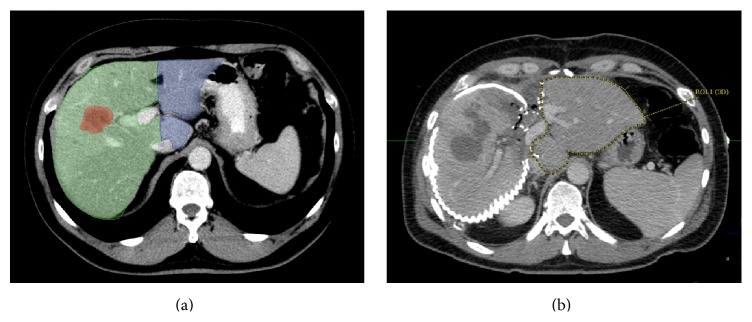
Pre- and postoperative CT-scan of the liver. Preoperative CT-scan depicting the tumor lesion (marked red) within the right liver lobe (a). Furthermore, the resected liver portion (marked green) and the resulting future liver remnant (FLR; marked blue) are shown. (b) CT-volumetry 10 days after the first step of ALPPS resulted in a significant increase of the FLR (marked by dotted yellow line). The extended right liver lobe (wrapped in a silicone matting) meanwhile showed signs of necrosis following ligation of the right portal vein.

The operation was started as to perform a right hepatectomy and intraoperative ultrasound was used to reevaluate the liver lesion. In contrast to the preoperative CT-scan, the known lesion in the right lobe of the liver was found to additionally infiltrate segment IVa, which was expected to be preserved; thus extended right hepatectomy would have been necessary to achieve tumor-free margins. Therefore, the liver was thoroughly reevaluated and deemed steatotic to a significant degree due to macroscopically visible fat lesions confirmed later by histology. Performing a right trisegmentectomy, an inadequate future liver remnant (FLR) would have been unavoidable. In conclusion, the decision was made to perform an ALPPS-procedure to enable safe resection of the ICC. Consequently, the hepatoduodenal ligament was dissected to isolate the right hepatic artery, portal vein, and bile duct. Then the right hepatic vein was isolated. After transection of the right portal vein, the hepatic parenchyma was dissected between segment II/III and segment IV. Small hepatic veins draining from the right liver into the vena cava likewise were transected. The right liver lobe was then wrapped in a silicone matting to prevent adhesions (Figure 1). The patient required no transfusions of packed red blood cells (pRBC) or fresh frozen plasma (FFP) and showed an uneventful postoperative recovery after the first step of the ALPPS-procedure with discharge from the intensive care unit (ICU) within 3 days. A retrospective volumetry of the FLR showed a volume of 291 cm^3^ representing approximately 21% of the total liver volume and a FLR/body weight ratio of 0.28 (Figure 1).

**Figure 2:**
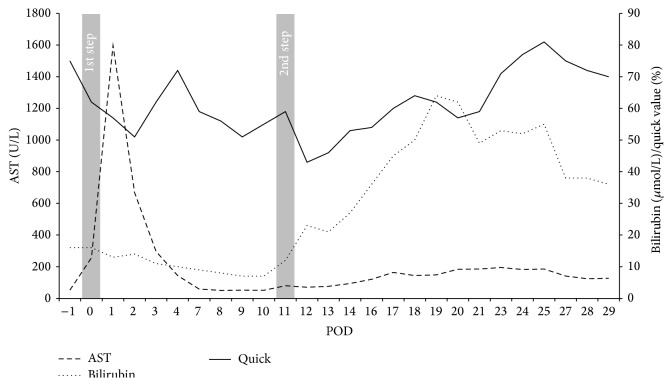
Postoperative course of AST, Bilirubin, and Quick value. Diagram depicting the courses of AST, Bilirubin, and Quick value following the first and second step of the ALPPS-procedure, respectively.

A CT-volumetry conducted on postoperative day (POD) 10 showed FLR volume of 591 cm^3^ (approximately 43% of the total liver volume) resulting in a volume increase of about 103% following step 1 of ALPPS. AST and ALT levels primarily were significantly elevated to 1598 U/I and 1429 U/I on POD 2 but constantly dropped thereafter and were 80 U/I and 118 U/I on POD 10, respectively (Figure 2). An adequate recovery of liver functions as monitored amongst others by the Quick value (Figure 2).

Thus, the second step of ALPPS was realized on POD 11. The mobilization of the liver turned out to be difficult due to strong adhesions in the hilar region. Finally, the silicon matting covering the liver was removed; right hepatic artery, right hepatic bile duct, and right hepatic vein were dissected. There was no major intraoperative complication; however, due to the strong adhesions two units of pRBC had to be transfused, but no further blood products such as FFP or platelets were required. The postoperative histology revealed the following tumor staging: T2, N1, L1, V1, Pn1, and R0.

In the postoperative course there were no major complications; again the patient could be discharged from ICU on POD 3. AST and ALT levels were only slightly elevated with no clinical signs of liver failure (Figure 2). Meanwhile Bilirubin was not elevated at any time point in between step 1 and step 2 of ALPPS; the latter was raised to 62 *μ*mol/L on POD 9 after the second operation. In the course, Bilirubin was seen to constantly drop but still being slightly elevated at the time of discharge (Figure 2). The Quick value again significantly dropped on POD 1 after the second step but recovered rapidly thereafter. Clinically, the only relevant impairment of the patient was sustained ascites and general edema (approximately 20 kg of weight gain) that efficiently responded to an intensified therapy with diuretics.

Prior to discharge, the Liver MAximum capacity (LiMAx) test based on intravenous application of methacetin [10] was performed and already revealed a normal liver function (LiMAx-value of 381 *μ*g/h/kg). The patient was discharged on POD 20 after completion of ALPPS. Due to the above-mentioned tumor staging an adjuvant chemotherapy with gemcitabine was recommended.

**Figure 3:**
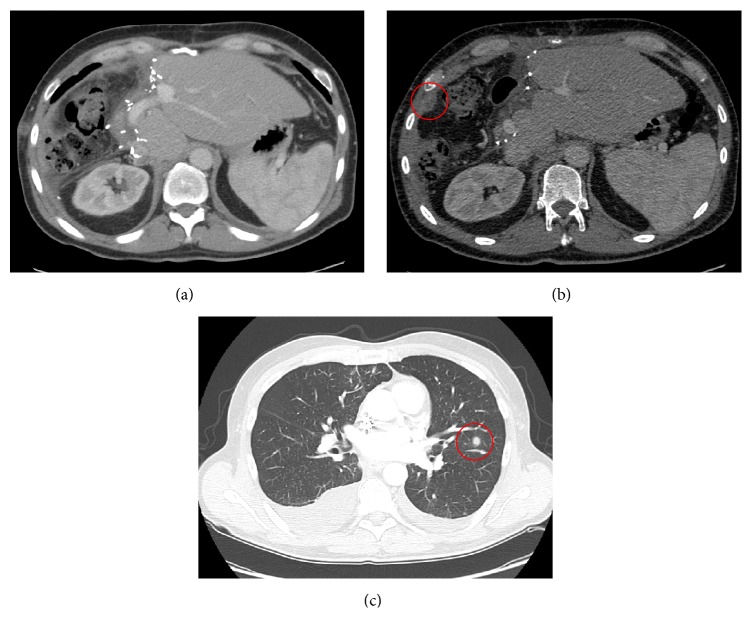
Follow-up CT-scan. Follow-up CT-scan approximately 2.5 months after liver resection showing the further volume increase of the liver remnant (a). Furthermore, examples of the novel extrahepatic tumor manifestations in terms of peritoneal carcinosis (b) as well as lung metastases (c) are shown (marked by red circles, resp.).

One month after discharge the patient presented in the outpatient clinic with thoracic discomfort. A follow-up CT-scan of the abdomen and chest was performed and revealed suspicious lesions in the left lung (at least 3 tumor lesions) and the peritoneum (at least 2 lesions suspicious of peritoneal carcinosis) (Figure 3). Consequently, a palliative chemotherapy was started with gemcitabine and cisplatin. However, the patient had to stop the chemotherapy due to severe side effects and passed away within 2.5 months after the tumor resection.

## 3. Discussion

First of all, the presented case indicates that an intraoperative conversion to an ALPPS-procedure might be a safe alternative approach to conventional liver resection when the FLR surprisingly seems to be insufficient. This is in line with the case series published by Truant and colleagues who reported seven successful ALPPS-procedures in which the decision was made intraoperatively without major additional complications [11].

However, ALPPS remains a relatively new technique and long-term results are still missing. Particularly in combination with the high morbidity and mortality observed in the first patients treated by ALPPS, preoperative planning including detailed imaging (usually within the last 3-4 weeks) and analysis of liver function seems essential to achieve best possible outcomes and perform a safe procedure. Even in case that the ALPPS-procedure represents an* ultima ratio* with intraoperative decision-making, it is necessary to obtain the informed consent from the patient prior to surgery.

In general, occlusion of the portal vein to increase the size of the FLR in order to lower the risk for postoperative small-for-size syndrome following extended liver resection is well established. Portal vein embolization (PVE) or portal vein ligation (PVL) usually are performed within 4 to 8 weeks prior to the planned liver resection. ALPPS combines PVL and complete parenchymal transection, followed by hepatectomy within 1 to 2 weeks [12].

In a recent meta-analysis PVE and PVL showed comparable results regarding increase of the FLR as well as morbidity and mortality of the patients. However, both techniques also showed around 20% of disease progression after the intervention [13]. Further, Nagino et al. reported an incidence of unresectability after PVE due to tumor progression in 12% of 150 cases treated for cholangiocarcinoma [14]. There is evidence that the ALPPS-procedure is advantageous to PVE and PVL in terms of significantly greater increase of the FLR and higher rates of completion of hepatectomy due to less tumor progression following portal vein intervention [12, 13]. However, mortality and morbidity seem higher among the ALPPS-treated patients compared to PVE and PVL [12, 15].

Extended liver resection—even applying measures to increase the FLR—always bears the risk of postoperative small-for-size syndrome. To date, the volume increase of the FLR (determined by CT-volumetry) and conventional laboratory values representing the liver function (e.g., liver enzymes, Quick value) are used for decision making to perform/complete the hepatectomy. Recently, the LiMAx-test was developed allowing further determination of liver function at the enzymatic level by methacetin kinetics. This tool successfully was applied to monitor volume increase of the FLR after portal vein occlusion as well as predict the postoperative outcome after liver resection [10, 16]. Hence, the advantage of an early completion of hepatectomy in case of ALPPS might further profit from application of the LiMAx-test: minimization of the risk of postoperative liver failure and disease progression might be achieved at the same time.

Regarding the treatment of cholangiocarcinomas the experience concerning ALPPS is still limited. It is suggested that there may be an association with unacceptably high rates of septic complications (60%) [17]. Regarding the subtype of hilar cholangiocarcinomas the combination of a stented biliary system and a cholestatic liver seems to be associated with worsened outcome and hence might be considered a contraindication for ALPPS [15]. Whether ICC represents a good indication for the ALPPS-procedure remains to be clarified. The first report of the international ALPPS registry presented about 8 patients with ICC which underwent ALPPS with a 90-day mortality rate of 13% and tumor recurrence in about 69% [18]. The survival at 1 year in the ALPPS registry was 73%. In comparison, patients without resection show a 1-year survival of only 26% [6]. It has to be kept in mind that, with ALPPS, a patient group with the highest possible risk of recurrence is treated surgically; most of those patients would never have had another chance for surgery. In conclusion, ALPPS should also be considered as a treatment option in well-selected patients with ICC.

The role of adjuvant chemotherapy after ALPPS represents another issue to be discussed, especially following resection of ICC. Currently, there is no general recommendation for adjuvant chemotherapy after the surgical therapy of ICC. Fisher et al. recommend adjuvant chemotherapy in the case of lymphovascular and perineural invasion due to the reduction of the overall survival in a retrospective study [19]. Further, Sur and colleagues reported that patients were found to significantly benefit from adjuvant chemotherapy if they had positive lymph nodes or positive resection margins [20]. The prospective ACTICCA-1 study to clarify the effect of an adjuvant chemotherapy is still ongoing. However, in our case the decision in favor of an adjuvant chemotherapy was made in our interdisciplinary tumor board due to the tumor staging and the fact that early tumor recurrence after ALPPS was also described for other tumor entities [21].

In conclusion, ALPPS should be considered a treatment option in well-selected patients with ICC, especially as a rescue procedure when an inadequate FLR is unavoidable. Adjuvant chemotherapy after ALPPS in case of ICC should always be discussed by an interdisciplinary tumor board.
